# Multimodal validation of focal enhancement in intracranial aneurysms as a surrogate marker for aneurysm instability

**DOI:** 10.1007/s00234-020-02498-6

**Published:** 2020-07-17

**Authors:** Naomi Larsen, Charlotte Flüh, Sylvia Saalfeld, Samuel Voß, Georg Hille, David Trick, Fritz Wodarg, Michael Synowitz, Olav Jansen, Philipp Berg

**Affiliations:** 1 Department of Radiology and Neuroradiology, University Hospital Schleswig-Holstein Campus Kiel, Arnold-Heller-Str. 3, Haus D, 24105 Kiel, Germany; 2 Department of Neurosurgery, University Hospital Schleswig-Holstein, Campus Kiel, Kiel, Germany; 3 Forschungscampus STIMULATE, University of Magdeburg, Magdeburg, Germany; 4 Department of Simulation and Graphics, University of Magdeburg, Magdeburg, Germany; 5 Institute of Fluid Dynamics and Thermodynamics, University of Magdeburg, Magdeburg, Germany; 6 Institute of Pathology, University Hospital Schleswig-Holstein, Campus Kiel, Kiel, Germany

**Keywords:** Intracranial aneurysm, Subarachnoid hemorrhage, Magnetic resonance imaging, Hemodynamics, Morphology, Histology

## Abstract

**Purpose:**

Circumferential enhancement on MR vessel wall imaging has been proposed as a biomarker of a higher risk of rupture in intracranial aneurysms. Focal enhancement is frequently encountered in unruptured aneurysms, but its implication for risk stratification and patient management remains unclear. This study investigates the association of focal wall enhancement with hemodynamic and morphological risk factors and histologic markers of wall inflammation and degeneration.

**Methods:**

Patients with an unruptured middle cerebral artery aneurysm who underwent 3D rotational angiography and 3T MR vessel wall imaging showing focal wall enhancement were included. Hemodynamic parameters were calculated based on flow simulations and compared between enhanced regions and the entire aneurysm surface. Morphological parameters were semiautomatically extracted and quantitatively associated with wall enhancement. Histological analysis included detection of vasa vasorum, CD34, and myeloperoxidase staining in a subset of patients.

**Results:**

Twenty-two aneurysms were analyzed. Enhanced regions were significantly associated with lower AWSS, lower maxOSI, and increased LSA. In multivariate analysis, higher ellipticity index was an independent predictor of wall enhancement. Histologic signs of inflammation and degeneration and higher PHASES score were significantly associated with focal enhancement.

**Conclusion:**

Focal wall enhancement is colocalized with hemodynamic factors that have been related to a higher rupture risk. It is correlated with morphological factors linked to rupture risk, higher PHASES score, and histologic markers of wall destabilization. The results support the hypothesis that focal enhancement could serve as a surrogate marker for aneurysm instability.

## Introduction

Intracranial aneurysms have a prevalence of up to 3% and generally harbor a low risk of rupture [1, 2]. Still, subarachnoid hemorrhage following rupture of an intradural aneurysm is associated with considerable mortality and morbidity [3]. Therefore, risk stratification of patients diagnosed with an unruptured intradural aneurysm is crucial, but optimal management remains controversial. Recently, published data suggest an association of wall enhancement in intracranial saccular aneurysms on MR vessel wall imaging with a higher risk for rupture [4–7]. Specifically, thick circumferential wall enhancement has been linked to a higher risk of rupture [4]. Moreover, wall enhancement probably identifies ruptured aneurysms in patients with subarachnoid hemorrhage and multiple aneurysms [8], and in AVMs, respectively [9]. In a prior study, inflammatory and degenerative wall changes were detected in a histologic analysis of unruptured aneurysms showing wall enhancement [10].

Focal enhancement has been reported to be associated with ruptured aneurysms and the rupture site [11, 12]. While low-flow conditions near the aneurysm wall have been described to coincide with focal enhancement [13], low wall shear stress (WSS) has been found to be associated with rupture sites [14]. Other studies related flow characteristics, such as low shear area (LSA), shear concentration index (SCI), oscillatory shear index (OSI), neck inflow rate (NIR), inflow concentration index (ICI), and WSS to aneurysm growth, rupture risk, and rupture [15–21].

Moreover, numerous morphological parameters including size ratio, undulation index, nonsphericity index, ellipticity index, aneurysm angle, aneurysm volume, neck size. and aspect ratio have been associated with aneurysm rupture [22–24].

Focal enhancement in unruptured aneurysms is frequently encountered on MR vessel wall imaging; still, its implication for patient risk stratification and management remains unclear. Hence, the goal of this study was to determine the correlation of focal enhancement with previously described risk factors in order to assess its suitability as a surrogate marker for a higher risk of rupture. To achieve this goal, we chose a multimodal approach: We investigated whether focal wall enhancement is colocalized with specific hemodynamic conditions related to a higher risk of rupture. Furthermore, the association with morphological parameters linked to rupture risk was assessed. Histologic data were analyzed to associate pathologic processes encountered during aneurysm evolution and wall remodeling with wall enhancement.

## Methods

### Patients

Ethics approval for this study was obtained from the local ethics committee. Written informed consent was obtained. We selected all patients from our institutional MR vessel wall imaging database with an unruptured middle cerebral artery aneurysm, who received high-resolution 3T MR vessel wall imaging showing focal wall enhancement and three-dimensional rotational angiography (3D-RA) from 7/2016 to 3/2019. Only cases with MRI and 3D-RA images of sufficient quality were included. Demographic data as age, gender, intake of acetylsalicylic acid and nicotine, hypertension, diabetes, and PHASES score were extracted from patient records. Histologic data were collected from a subset of patients who underwent microsurgical clipping of the aneurysm. In our institution, the neurosurgeon and interventional neuroradiologist determine in an interdisciplinary consensus-based decision-making process whether to recommend treatment of the aneurysm and the preferred method.

### MR vessel wall imaging

MR vessel wall imaging was acquired on a 3T MR scanner (Achieva, Philips Healthcare, Best, The Netherlands) equipped with a 32-channel head coil. The protocol comprised a T1-weighted black blood 3D variable refocusing flip angle sequence (VISTA) (TE/TR, 27/700 ms; matrix, 268 × 332; field of view, 200 × 250 × 160 mm; voxel size, 0.75 × 0.75 × 0.75 mm; acquisition time, 4 min 45 s) and a TOF-angiography; both sequences were acquired before and after administration of 0.1 mmol/kg gadoterate meglumine (Dotarem, Guerbet, Villepinte, France).

Contrast enhancement of the aneurysm wall was visually assessed on multiplanar reformatted postcontrast 3D T1 black blood images.

Focal wall enhancement was defined as a hyperintense signal adjacent to the black blood lumen or wall-adherent thrombus with at least one clearly discernible unenhanced wall segment of any extent. The presence of a double-layer appearance indicative of thrombus, defined as a non-enhancing, wall-adjacent structure bordered by an enhancing layer on both the luminal and the wall-adjacent surface, was documented.

Two neuroradiologists (N.L., F.W.), each with at least 9 years of experience in neurovascular imaging, who were blinded for clinical and hemodynamic data, independently assessed the aneurysm wall. Disagreement between the readers was solved by consensus.

### Digital subtraction angiography

Digital subtraction angiography (DSA) was performed under local anesthesia on a biplane flat panel DSA unit (Allura Xper FD 20/10, Philips, Best, The Netherlands) via a femoral access. 3D-RA was performed with a selective contrast injection of 20 ml Imeron 300 (Iomeprol, Bracco Imaging, Milan, Italy) in the ipsilateral internal carotid artery at a flow rate of 2 ml/s. The dataset was transferred to a dedicated workstation (XtraVision, Philips, Best, The Netherlands) and reconstructed with a voxel size of 0.27 × 0.27 × 0.27 mm^3^.

### Vessel segmentation and image co-registration

**Fig. 1:**
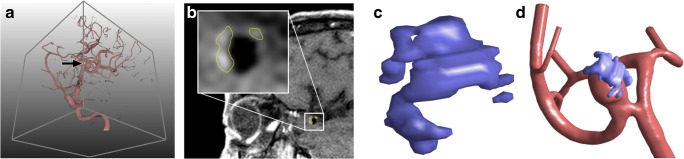
**A** Illustration of a 3D rotational angiography (3D-RA) dataset of a middle cerebral artery aneurysm (arrow). **B** Corresponding MRI dataset with yellow contours delineating enhanced regions. **C** 3D model of the enhanced regions. **D** Co-registered 3D-RA aneurysm surface mesh and 3D MRI enhancement surface mesh

Segmentation of vessel surfaces from 3D-RA datasets and co-registration to MRI was carried out using MeVisLab 2.7 (MeVis Medical Solutions AG, Bremen, Germany). A threshold-based segmentation approach was applied to the 3D-RA images, followed by marching cubes to obtain a triangulated surface mesh. Enhanced regions were manually delineated in black blood MR images using the contour segmentation objects library of MeVisLab. The enhancement mask was converted into a triangle surface mesh as well. Finally, co-registration utilizing a rigid multi-resolution registration [25] with a Quasi-Newton optimizer within MeVisLab yielded transformation matrices for each patient’s dataset. Applying the transformation matrices to the manually segmented enhancement regions allowed for the combination with the vessel surface (see Fig. 1). The percentage of the aneurysm surface area covered by enhanced regions was determined for each aneurysm (enhancement area EA) and utilized as a quantitative measure for the extent of enhancement.

### Hemodynamic simulations

Based on the segmented aneurysm surfaces, hemodynamic simulations derived from 3D-RA datasets were carried out in all cases using STAR-CCM+ 13.04 (Siemens PLM Software Inc., Plano, TX, USA). For spatial discretization a base size of Δ*x* = 0.08 mm was chosen leading to 1.2 to 3.1 million polyhedral and prism elements in total depending on the considered vasculature. At each inlet, a time-dependent flow waveform was applied obtained from PC-MRI measurements in a healthy volunteer [26]. Flow-splitting at all outlet cross-sections was defined according to the recent model by Chnafa et al. [27]. Blood was treated as an incompressible (*ρ* = 1055 kg/m^3^) and Newtonian (*η* = 4 mPa s) fluid, while the flow was assumed to be laminar. Three cardiac cycles were simulated, while only the last one was considered for analysis.

Quantification of shear-related parameters was conducted for enhanced and non-enhanced wall segments separately, including time-averaged wall shear stress (AWSS), normalized wall shear stress (nWSS), maximum oscillatory shear index (maxOSI), and low shear area (LSA). Mean neck inflow rate (meanNIR), inflow concentration index (ICI), and shear concentration index (SCI) were determined for entire aneurysms [28].

### Semi-automatic extraction of morphological parameters

In each aneurysm, a semiautomatic neck curve reconstruction was performed. First, four points in the neck region of the aneurysm were determined following a manual one-click selection of the aneurysm. Based on these points, a neck curve was created and the ostium plane derived. For a detailed description of the computational process, see Saalfeld et al. [29]. Utilizing the neck curve and ostium plane, the following morphological parameters were automatically extracted: aneurysm surface area (A), aneurysm volume (V), ostium area1 (OA1), ostium area2 (OA2), maximum diameter (Dmax), maximum height (Hmax), maximum width perpendicular to Hmax (Wmax), maximum height perpendicular to the ostium plane (Hortho), maximum width parallel to the ostium plane (Wortho), maximum neck curve diameter (Nmax), average neck curve diameter (Navg), aspect ratio 1 (AR1), aspect ratio 2 (AR2), volume of the convex hull (Vch), surface area of the convex hull (Ach), ellipticity index (EI), non-sphericity index (NSI), undulation index (UI), and aneurysm tilt angle *γ* [29].

### Aneurysm clipping and histologic analysis

Microsurgical clipping of the aneurysms was performed via a pterional-transsylvian approach.

Preoperative, postoperative, and intraoperative management of the patients was performed according to institutional standards, including preoperative and postoperative DSA of the intracranial vessels, and, in most cases, intraoperative angiography with indocyanine green. After clipping of the aneurysm, the aneurysm sac was removed.

Histologic analysis was performed as described before [30]. In brief, formalin-fixed and paraffin-embedded resection specimens were cut into 2.5-μm-thin tissue sections. Slides were stained either with hematoxylin and eosin, or using rabbit anti-MPO polyclonal antibodies (1:1000, DAKO, Glostrup, Denmark) after antigen retrieval with ER2 (EDTA-buffer bond pH 8.9), or a monoclonal antibody against CD34 (Beckman Coulter, Clone QBEnd10, 1:700) without antigen retrieval using the autostainer Bond Max System (Leica Microsystems GmbH, Wetzlar, Germany).

### Statistical analysis

Statistical analysis was performed in RStudio (RStudio v1.2.1335 with R v3.5.1, RStudio Inc., Boston, USA). Normally distributed variables are presented as mean (range), non-normally distributed continuous variables as median (range) or median (standard deviation), and categorical variables in the form of percentage distributions. A Shapiro–Wilk test was employed to determine, if a continuous variable was normally distributed. Non-normally distributed data were nonlinearly transformed before they were included in univariate analysis. Hemodynamic parameters were analyzed using the Wilcoxon signed-rank test for comparison of enhanced regions and the entire aneurysm surface, and the Mann–Whitney *U* test for comparison of the enhancement area EA in aneurysms with and without histologic markers of inflammation and wall degeneration. Pearson’s correlation test was used to assess the relationship of hemodynamic and morphological parameters with the enhancement area EA. Only parameters that were significantly associated with EA in univariate analysis were entered into a linear regression model in order to restrict the number of variables included in multivariate analysis. Furthermore, since some of the morphological variables have been reported to show a strong degree of correlation [29], only the variable with the lowest *P* value was selected for multivariate analysis. A *P* < 0.05 was considered significant in univariate and multivariate analyses.

## Results

Twenty-one patients with 22 aneurysms were included; 71% were located on the right side. Clinical data were available for all included patients. The interrater agreement concerning focal enhancement was rated as substantial (Cohen’s *κ* = 0.742). Demographic information of the patients is summarized in Table 1. The PHASES score was positively correlated with EA (*P* = 0.0275). An association of gender, age, hypertension, diabetes, ASA and statin intake, smoking, and aneurysm location with EA could not be demonstrated.

**Table 1:** Demographic characteristics

Parameter	
Gender ratio (% of female)	76%
Age (mean (range))	60 years (48–84)
Hypertension	86%
Diabetes	14%
ASA	24%
Statin	14%
Nicotine	57%
PHASES (median (mode))	6 (6)

Demographic data of all included patients

*ASA* acetylsalicylic acid, *PHASES* population, hypertension, age, size of aneurysm, earlier subarachnoid hemorrhage from another aneurysm, site of aneurysm

Microsurgical clipping with subsequent histologic analysis of the resected aneurysm sac was conducted in 9 patients. The surgical indication was based on the patient’s preference in 5 cases, and on aneurysm morphology (wide-necked aneurysm) in 4. Features of all aneurysms alongside histologic data are listed in Table 2.

**Table 2:** Size, enhancement area, and histologic features of all aneurysms

Aneurysm no.	Size (mm)	Enhancement area (%)	Histologic parameter				
			MPO	CD34	Vasa vasorum	Lipid deposition	Thrombus
1	8	2.13					
2	9	0.97					
3	11	22.51	+			+	+
4	9	6.49	+		+	+	
5	7	31.39	+				
6	7	0.49					
7	10	43.09	+		+		+
8	6	7.30					
9	5	33.23			+		
10	9	32.79					
11	8	28.27					
12	11	73.64					
13	7	29.79					
14	8	26.55					
15	3	32.40					
16	15	58.60					
17	23	23.40					
18	8	5.43					
19	5	41.74					
20	7	33.65					
21	8	25.22					
22	7	30.11					
Median	8	26.55					

Size (as manually measured on 2D angiographic images), enhancement area, and histologic features of all aneurysm. Histologic specimens were available from aneurysms 1–9

### Wall enhancement and blood flow evaluation

Flow simulation and calculation of hemodynamic variables were successful in all patients. A qualitative observation of the blood flow revealed that flow structures perpendicular to the main flow direction occurred in the vicinity of the enhancement zones. In cases with an increased aspect ratio, it was observed that these flows move sideward through the aneurysm sac instead of following the global flow direction. Additionally, it is important to highlight that enhancement appeared predominantly at the sides of the wall and was only present at the aneurysm dome in one case. Thus, the entering flow-jet and the corresponding impingement zone (which causes increased shear stresses on the luminal surface) were not associated with the enhanced wall segments.

To further quantify the flow behavior along the aneurysmal lumen comparing enhanced and non-enhanced regions, three shear-related parameters were assessed.

AWSS was significantly lower in enhanced wall segments (3.74 Pa (3.91 Pa)) compared with the entire aneurysm wall (7.76 Pa (4.78 Pa)) (*P* < 0.0001), while maxOSI decreased from 0.45 (0.05) to 0.37 (0.14) (*P* < 0.0001). In the analysis of LSA, values were significantly higher in enhanced segments (68.09% (34.36%)) compared with the entire aneurysm (56.91% (30.34%)) (*P* < 0.0001).

There was no significant correlation of inflow-related hemodynamic parameters or SCI with EA. There was a trend towards higher LSA in aneurysms with a larger extent of enhancement (*P* = 0.08222). For a summary of the analysis of hemodynamic and morphologic parameters, see Table 3.

**Table 3:** *P* values from univariate analyses of the relationship of enhancement (enhancement area EA) with hemodynamic and morphologic parameters

Hemodynamic parameter	*P* value	Morphologic parameter	*P* value
Time-averaged wall shear stress (AWSS)	0.5696	Aneurysm surface area (A)	0.06235
Normalized WSS (nWSS)	0.8343	Aneurysm volume (V)	0.06894
Maximum oscillatory shear index (maxOSI)	0.217	Ostium area1 (OA1)	0.06962
Low shear area (LSA)	0.08222	Ostium area2 (OA2)	0.04929*
Mean neck inflow rate (meanNIR)	0.9488	Maximum diameter (Dmax)	0.02093*
Inflow concentration index (ICI)	0.1237	Maximum height (Hmax)	0.0421*
Shear concentration index (SCI)	0.6168	Maximum width (Wmax)	0.1363
		Maximum height perpendicular to the ostium plane (Hortho)	0.1349
		Maximum width parallel to the ostium plane (Wortho)	0.04025*
		Maximum neck curve diameter (Nmax)	0.2294
		Average neck curve diameter (Navg)	0.1107
		Aspect ratio 1 (AR1)	0.7308
		Aspect ratio 2 (AR2)	0.8133
		Volume of the convex hull (Vch)	0.04633*
		Surface area of the convex hull (Ach)	0.04644*
		Ellipticity index (EI)	0.008209*
		Non-sphericity index (NSI)	0.04134*
		Undulation index (UI)	0.03966*
		Aneurysm tilt angle *γ*	0.7401

*Statistically significant (*P* < 0.05)

### Wall enhancement and morphologic parameters

OA2, Dmax, Hmax, Wortho, Vch, Ach, EI, NSI, and UI were significantly correlated with EA in univariate analysis (Table 3). EI showed the lowest *P* value and was entered alongside Dmax into multiple regression analysis, the remaining variables were not considered due to collinearity as described before [29]. EI remained significantly associated with EA (*P* = 0.0395).

### Histologic features

**Fig. 2:**
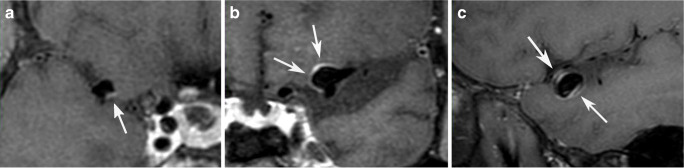
Post-contrast 3D T1 black blood images showing **A** aneurysm with a low extent of wall enhancement and **B** aneurysm with substantial enhancement of the wall. **C** Double layer appearance in an aneurysm with wall-adjacent thrombus that was histologically confirmed

**Fig. 3:**
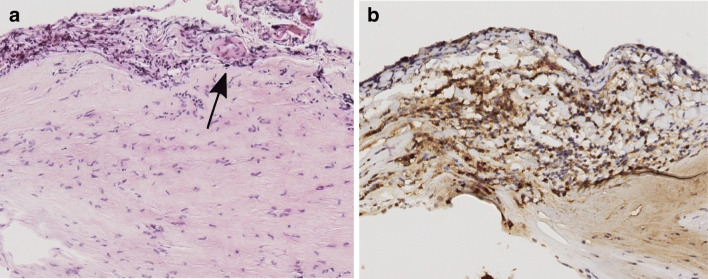
Representative photomicrographs showing **A** vasa vasorum (arrow) in the adventitial layer (hematoxylin and eosin stain, original magnification × 10), and **B** numerous myeloperoxidase-expressing cells staining brown (myeloperoxidase stain, × 10)

Four aneurysms stained positive for MPO-expressing cells. In three aneurysms, vasa vasorum were present. CD34-positive neovessel endothelium could not be detected in any of the aneurysms. Representative cases are illustrated in Figs. 2 and 3. In this subset of patients, the presence of one or two of these histologic markers was significantly associated with a larger extent of wall enhancement (*P* = 0.03175).

Wall-adherent thrombus was detected on MR vessel wall imaging in two aneurysms, and intraluminal wall adherent thrombus was confirmed by histologic analysis. Enhancement was visible both on the luminal surface of the thrombus and the adjacent wall, leading to a double-layer appearance (Fig. 2c).

Extracellular lipid deposition was detected in two aneurysms. Hyalinosis, fibrosis, and calcification were common features in most aneurysms.

## Discussion

### Focal enhancement, hemodynamic parameters, and morphology

In this study, focal enhancement was colocalized with low AWSS, low maxOSI, and high LSA in agreement with results recently published by Xiao et al. and Khan et al. [13, 31], confirming the assumption that enhanced regions possibly indicate the presence and localization of low-flow conditions in an aneurysm.

This observation adds to the assumption that the extent of focal enhancement depends on the prevalence of low-flow conditions. Furthermore, it seems likely that there is a continuous spectrum of the extent of wall enhancement correlated to a hemodynamic environment promoting wall destabilization that has to be considered when assessing rupture risk, rather than an either-or presumption that is implied when dichotomizing unruptured aneurysms into either enhancing (and thus at higher rupture risk) or non-enhancing. There was no significant correlation of AWSS, maxOSI, and LSA with EA when computed for whole aneurysms. Still, there was a trend towards higher LSA in aneurysms with a larger extent of enhancement (*P* = 0.08222). This could be explained by the fact that the percentage of the aneurysm surface that was covered by enhancement was relatively low in the aneurysms included in this study (median 27%; see Table 3). Future longitudinal studies focusing on quantification of aneurysm wall enhancement on MR vessel wall imaging and correlated clinical parameters are warranted.

Numerous studies have described morphologic characteristics associated with a ruptured state, such as larger aneurysm size and greater aspect ratio [15, 17, 22, 32, 33]. Yet, a retrospective longitudinal analysis conducted by Leemans et al. yielded no significant differences in hemodynamic and morphological parameters at baseline imaging in aneurysms that had grown versus stable aneurysms [23]. Morphological and hemodynamic analysis in cases where longitudinal data are not available might therefore not be able to sufficiently discriminate between stable and unstable aneurysms. In our patient cohort, aneurysm enhancement was positively correlated with hemodynamic, morphologic, and histologic markers that have been linked to an unstable state, which supports the assumption that wall enhancement might reflect the complex interactions and multifactorial pathologic processes in aneurysm evolution leading to wall destabilization, while a direct causal relation to either of these factors cannot readily be assumed.

### Low-flow conditions, thrombi, and histologic findings

In a comprehensive study linking macroscopic wall appearances to specific hemodynamic conditions, Cebral et al. found low AWSS to be associated with macroscopically hyperplastic and atherosclerotic wall segments [34]. Concordantly, we observed histologic markers of inflammatory and remodeling processes in aneurysms with a greater extent of focal wall enhancement, suggesting that these pathologic conditions are more abundant in conjunction with low-flow conditions.

Moreover, rupture sites of intracranial aneurysms have been associated with low wall shear stress [14, 34–36] and focal enhancement [11, 12]. Still, a direct association of low-flow areas and possible future rupture site cannot directly be deduced from the results of the present study, because local matching of histologic findings and enhancements areas was not conducted.

Recently, Sato et al. published their results from a 7T vessel wall MRI study describing the double-layer appearance in thrombosed aneurysms and linking them to histologic signs of inflammation, in accordance with our results [37].

Matsushige et al. described focal enhancement as possibly attributable to the presence of thrombotic material at rupture sites [11]. Another study attempted to categorize aneurysm wall types and found thrombosis as a feature of categories with a higher proportion of ruptured aneurysms [38]. These observations add to the assumption that slow blood flow and thrombus formation might play a substantial role in wall destabilization and can be visualized with MR vessel wall imaging.

Still, the observation that focal enhancement, albeit to a lesser extent, is also encountered in aneurysms lacking histologic signs of wall inflammation, possibly reflects the proposed theory that there are different pathways of wall destabilization in intracranial aneurysms [39]. Substantial wall enhancement might primarily reflect the inflammatory pathway of wall remodeling, while the sensitivity for the mural-cell-mediated pathway could be diminished.

Cornelissen et al. assumed that wall enhancement is not causally linked with inflammatory processes but rather attributable to method-inherent failure of blood signal suppression in low-flow conditions [40, 41]. The results of our study suggest that inflammatory changes probably add to the extent of wall enhancement, and that even without convincing proof of a causal link, wall enhancement indicates the presence of a hemodynamic environment promoting inflammatory and degenerative processes and might therefore still serve as a surrogate marker for aneurysm wall instability.

The extent of enhancement seems to depend on the sequence employed in the vessel wall MR protocol, with the T1 TSE black blood sequence like the one used in our study possibly being more sensitive for the detection of slow blood flow compared with motion-sensitized driven equilibrium (MSDE)-prepared sequences [42].

This study has several limitations. The retrospective study design might imply a selection bias towards patients who underwent DSA and were potentially pre-selected for therapy due to their risk profiles. Additionally, histologic analysis was exclusively performed in patients who underwent clipping, possibly inducing bias towards aneurysms with morphologies unfavorable for endovascular therapy. Histological data were only available in nine aneurysms. Microsurgical clipping provides only specimens of the aneurysm dome; neck segments under the clip cannot be retrieved. The small sample size precluded subgroup analysis of histologic features with adequate power. We chose to restrict our analysis to middle cerebral artery aneurysms in an effort to exclude a possible heterogeneity in aneurysm phenotype caused by different pathogenetic mechanisms potentially underlying the evolution and progression of aneurysms in different locations [43]. Additionally, an overestimation of the neck size using 3D imaging techniques can occur compared with 2D images, but careful segmentation using advanced algorithms was applied [44, 45]. Finally, no patient-specific inflow conditions were available for the blood flow simulations. However, a comparison between enhanced regions and the whole aneurysm remains feasible.

## Conclusion

Focal wall enhancement in unruptured intracranial aneurysms is colocalized with hemodynamic factors that have been related to a higher risk of rupture. It is correlated with morphological factors linked to rupture risk, a higher PHASES score, and histologic markers of wall destabilization. The results support the hypothesis that focal enhancement could serve as a surrogate marker for aneurysm instability.

## Data Availability

The datasets analyzed in the current study are available from the corresponding author on reasonable request.
